# PuraStat RADA16 Self-Assembling Peptide Reduces Postoperative Abdominal Adhesion Formation in a Rabbit Cecal Sidewall Injury Model

**DOI:** 10.3389/fbioe.2021.782224

**Published:** 2021-12-10

**Authors:** Eun Seok Gil, Elton Aleksi, Lisa Spirio

**Affiliations:** 3-D Matrix Inc., Newton, MA, United States

**Keywords:** self-assembling peptide, nanofiber, RADA16, PuraStat, abdominal adhesions, cecal sidewall injury, wound healing, hydrogel

## Abstract

**Objective:** To evaluate the effect of PuraStat (2.5% RADA16) administration on postoperative abdominal adhesion formation in an *in vivo* model.

**Methods:** Anesthetized New Zealand white rabbits underwent cecal sidewall abrasion surgery in which the cecal serosa and juxtaposed parietal peritoneum were abraded after access through an abdominal midline incision. Eight animals were randomized to receive PuraStat administration at the interface of the injured tissues before incision closure, and five animals served as untreated controls. Treated animals received 3–12 ml PuraStat solution per lesion. Animals were sacrificed 14 days after surgery and examined for adhesion formation at the wound site.

**Results:** At study terminus, adhesions were identified in 90% (9/10) of abraded cecum/peritoneal wound sites in untreated controls versus 25% (4/16) of PuraStat-treated sites (*p* = 0.004). Mean ± SD Total Adhesion Score (average of the values for extent + strength of the adhesion in both defects per animal; maximum score = 14 points) was significantly 76% lower in PuraStat-treated animals (2.0 ± 3.0 points) compared to untreated controls (8.2 ± 1.9 points) (*p* = 0.029). Mean adhesion coverage area of wound sites was 79% lower in PuraStat-treated animals than controls (*p* < 0.001), and mean adhesion durability was 72% lower in PuraStat-treated animals versus controls (*p* = 0.005). Remnant hydrogel was observed at the wound sites of 75% of treated animals at postoperative Day 14.

**Conclusion:** PuraStat treatment has a positive protective effect in the cecal sidewall injury model, and significantly reduces abdominal adhesion formation at the interface of the injured cecum and overlying peritoneal sidewall defect.

## Introduction

Abdominal adhesions are bands of scar-like tissue that form between two or more organs or between organs and the peritoneal wall. Postoperative adhesion formation is the most frequent complication of abdominal and pelvic surgery (Tabibian et al., 2017), with a reported prevalence ranging from 54–93% in individuals with a history of prior abdominal surgery (Weibel and Majno, 1973; Menzies and Ellis, 1990; Ellis et al., 1999; Okabayashi et al., 2014). Although most patients with intra-abdominal adhesions remain asymptomatic, adhesions confer a life-long risk for patients, especially if they require subsequent open abdominal surgery and their practitioner is unaware of the presence of scarring (ten Broek et al., 2013a). Adhesions can complicate repeated abdominal surgery and extend operative time, and can cause symptomatic small bowel obstruction, female infertility, and chronic pain (ten Broek et al., 2013b). Despite recent advances in surgical techniques designed to better segregate tissues and keep a clear surgical field, no truly reliable clinical strategy exists to prevent postoperative adhesions (Moris et al., 2017). We hypothesized that targeted administration of biocompatible, hydrogel-forming, self-assembling peptides could be a useful intraoperative approach for reducing adhesion formation between the peritoneum and underlying intestines during open abdominal surgery.

RADA16 is a synthetic 16-amino acid peptide that remains solubilized as discrete β-sheet nanofibers in acidic aqueous solutions, which spontaneously begin cross-linking into complex three-dimensional (3D) hydrogel matrices within seconds after exposure to the physiological pH of blood, interstitial fluid, and lymph (Edwards-Gayle and Hamley, 2017; Zhang, 2017; Lee et al., 2019; Wang et al., 2019; Sankar et al., 2021). RADA16 has been shown to be an effective topical hemostatic agent, and a commercial formulation, PuraStat®, (Instructions for Use, 2014; 3-D Matrix Europe SAS, Caluire-et-Cuire, France) is currently CE marked in Europe, cleared in the US, approved in Japan, and licensed in Australia to control intra-operative bleeding in various surgical procedures including cardiovascular, gastrointestinal (GI), and/or otorhinolaryngological applications (Sankar et al., 2021; 3-D Matrix Inc. and Needham, 2014; United States Food and Drug Administration 2021). In some countries, PuraStat is also approved for treating delayed postoperative bleeding. A related RADA16-based product is PuraSinus^®^ (also a 2.5% solution), cleared by the US FDA in 2019 for use in nasal surgery and trauma repair as an intraoperatively applied hemostatic wound dressing that also prevents adhesion formation and supports wound healing (United States Food and Drug Administration, 2019; 3-D Matrix Europe SAS, 2019). Application of RADA16 formulations is straightforward, with syringe and nozzle or catheter delivery allowing easy administration of the viscous solution to difficult-to-reach target sites. The transparent nature of the *in situ* formed hydrogel in the body allows clear visualization of the surgical field and facilitates defect evaluation before closure. Given RADA16’s demonstrated ability to prevent adhesion formation in the nasopharyngeal mucosa after endoscopic surgery (Lee et al., 2017; Wong et al., 2020), we evaluated its utility as a surgical device for preventing adhesion formation following intra-abdominal open surgery.

The rabbit cecal sidewall abrasion model is an established *in vivo* approach for evaluating interventions for preventing postoperative abdominal adhesions. The rabbit model provides a reliable mammalian model that has previously been used to explore the anti-adhesion forming potential of, for example, polyethylene glycol/polylactic acid films (Rodgers et al., 1998), hyaluronan hydrogel (Yeo et al., 2007), anti-proliferative agents (Cooper et al., 2007), and topical hemostats (Hermans et al., 2012). Briefly, the cecum of anesthetized rabbits is accessed through a midline abdominal incision. Bleeding wounds are mechanically created on the cecal serosa by abrasion using dry gauze and on the overlying parietal peritoneum by shallow scalpel excision/scraping. The wound interface is either left untreated or is treated with the test article, followed by abdominal closure and examination for adhesion formation after an appropriate healing period. We used this model to compare adhesion development in animals that had the tissue interface at the wound site treated with PuraStat (2.5% RADA16) to animals that received no intervention.

## Methods and Materials

### PuraStat Device

**FIGURE 1:**
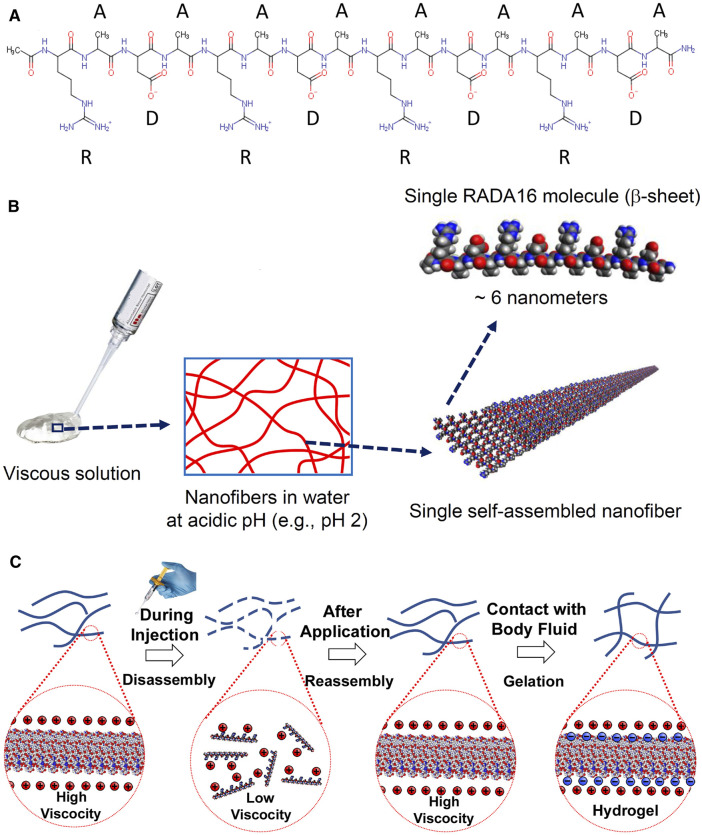
Chemical structure and self-assembly of RADA16 into higher-order hydrogels. **(A)**: The RADA16 peptide has 16 amino acids organized as repeated 4-amino acid sequences containing “R” (positively-charged arginine), “A” (hydrophobic alanine), and “D” (negatively-charged aspartic acid) residues. **(B)**: RADA16 undergoes spontaneous and revisable self-assembly in acidic solutions to generate nanofibers. RADA16 molecules with β-sheet conformation interact through face-to-face hydrophobic interactions and edge-to-edge hydrogen bonding to form layered and extended nanofibers, ∼ 6 nm wide. These extracellular matrix-like nanofibers form a viscous and transparent aqueous solution at a relatively low concentration range (e.g., 0.1–2.5% weight/volume). **(C)**: Illustration of RADA16 structure and properties as it is applied to and gels on a wound site. Acidic aqueous solutions of RADA16 are viscous and exhibit thixotropic disassembly/reassembly, which allows their easy administration to wound sites through catheters and syringes, with viscosity returning immediately after administration. Upon contact with the physiological pH of body fluids including blood, lymph and interstitial fluid, the surface net charges of RADA16 nanofibers become zero resulting in the physical crosslinking by hydrophobic interactions between neighboring RADA16 nanofibers, so that RADA16 solution forms *in-situ* hydrogels on the wound site and act as a physical adhesive that is hemostatic and supports wound healing. Adopted from Sankar et al., *Front. Bioeng. Biotechnol*. 2021; doi: 10.3389/fbioe.2021.679525, in accordance with Creative Commons Attribution License CC-BY.

The PuraStat Device (3-D Matrix, Ltd., Tokyo, Japan) is a 2.5% RADA16 formulation that is CE-marked as Class III medical devices for hemostatic use in humans. It is indicated as adjunctive hemostatic supplements to intraoperative ligation and suturing, to control exudative bleeding from small blood vessels and parenchyma of solid organs, at vascular anastomoses, and from small vessels of the GI tract mucosa following endoscopic and laparoscopic tissue resection (3-D Matrix Europe SAS, 2019). It is also licensed for similar use in Australia. In the US and Europe, PuraStat is cleared/approved for intra-operative use and to treat delayed bleeding after GI endoscopic surgery (United States Food and Drug Administration, 2021). No systemic adverse events have ever been reported when using RADA16 formulations for cleared/approved intra-operative uses. RADA16 is a synthetic oligomeric self-assembling peptide constructed with four repeating amino acids (arginine-alanine-arginine-aspartic acid)_4_ (Figure 1A). In the aqueous PuraStat formulation (2.5% RADA16, pH ≈ 2), the peptide exists as a viscous solution of nanofibers that are physically cross-linked into a hydrogel when exposed to the pH and ionic conditions of physiological environments (Figures 1B,C). PuraStat formulations of RADA16 also exhibit thixotropic reorganization upon exposure to shearing forces that allow it to precisely conform to the interface of two juxtaposed mobile tissues (Figure 1C). This feature facilitates adaptive retention when applied to wound sites involving two different adjacent tissues with potential mobility relative to each other.

### Animals

13 female nulliparous New Zealand White rabbits aged ≈6 months were purchased from Robinson Services, Inc. (Mocksville, NC), and acclimated for 5 days before experimentation. Climate was maintained at 61–72°F with relative humidity 30–70%, with a 12 h/12 h light/dark cycle, and food and water were provided *ad libitum*. Conditions were overseen by the NAMSA Contract Research Organization’s (Northwood, OH) Institutional Animal Care and Use Committee (IACUC) and conformed to the “*Guide for the Care and Use of Laboratory Animals, 8th edition*” (National Research Council, 2003). NAMSA is an AAALAC International accredited facility and is registered with the U.S. Department of Agriculture.

### Experimental Cecal Sidewall Injury Model

On the day of surgery, animals were injected subcutaneously with 0.05 mg/kg of buprenorphine, and a fentanyl patch (25 μg/h) was applied to one ear. Animals were anesthetized with an intramuscular injection of combination of ketamine hydrochloride and xylazine (34 mg/kg + 5 mg/kg) dosed at 0.6 ml/kg. Veterinary ophthalmic ointment was applied to both eyes to prevent corneal drying, and a prophylactic dose of enrofloxacin (5.0 mg/kg) was administered intramuscularly. Abdomens were clipped, disinfected with germicidal soap and 70% isopropyl alcohol, and the surgical site was marked with povidone iodine. General anesthesia was maintained using metered isoflurane inhalation. Vital signs (temperature, heart rate, SPO_2_) were monitored throughout the surgical procedure.

The rabbit cecal sidewall model is used to evaluate interventions for intra-abdominal post-surgical adhesions. This model predictably and quickly generates robust abdominal adhesion formation, with study endpoints typically selected between 7–30 days (Rodgers et al., 1998; Cooper et al., 2007; Yeo et al., 2007; Hermans et al., 2012). By random selection, eight animals were assigned to the PuraStat group, and five animals served as controls that underwent surgery but did not receive test article treatment. An ≈12-cm skin incision was made along the ventral abdomen midline, beginning 6 cm caudal to the xiphoid process. The abdominal wall was opened by incising along the linea alba. The entire cecum was exteriorized and abraded by wiping the entire serosal surface with a sterile dry gauze sponge until punctate bleeding was observed. The cecum was then repositioned in the abdomen, and bilateral defects measuring ≈2 × 4.5 cm were made to the parietal peritoneum over the abdominal sidewall. Defects were made ≈4 cm lateral to the midline incision and ≈7–9 cm caudal to the xiphoid process. An approximate 2 × 4.5-cm window of peritoneum was excised by sharp dissection and the muscle wall was disrupted by scraping the area with a scalpel blade to produce surgeon-adjudicated oozing and low-volume bleeds across all wound sites. All wound sites were irrigated with sterile saline, followed by PuraStat application in test animals that covered and coated the abraded sidewall sites and overlying peritoneum. The control animals were not treated with PuraStat. The sidewall and cecum were returned to normal positioning and the abdominal wall was closed by simple continuous suturing with 4-0 absorbable suture. Subcutaneous tissue was also closed with by simple continuous suturing. The skin was closed with stainless steel wound clips.

Animals were moved to a recovery area and placed on a heat source until they recovered from anesthesia. After sternal recumbency was achieved, animals were fitted with an Elizabethan collar and returned to their cage. Buprenorphine (0.05 mg/kg, SC) was administered 6 h after the initial pre-surgical dose. Enrofloxacin (5.0 mg/kg, IM) was administered at the end of the day of surgery and then twice/day for the first 2 days after surgery. All animals exhibited reduced, loose, or no feces following surgery and were provided food supplements and/or hay to stimulate recovery; all incidences were resolved by Day 14 study termination. Elizabethan collars were removed once the incision healed. Body weights were recorded prior to implantation, at Day 7, and at Day 14 termination.

### Terminal Procedures and Outcome Measures

At 14 (±1) days after surgery, animals were weighed and euthanized by an intravenous injection of sodium pentobarbital solution. The peritoneal cavity was opened and the viscera were examined by a staff veterinarian. To maintain consistency in lesion/adhesion grading, the same veterinarian conducted all evaluations. Defect sites of each animal were photographed, and each site was examined for adhesion formation. Adhesions were graded for extent and strength, per the criteria in Tables 1, 2, using a modification of a previously detailed Adhesion Area and Strength scoring system (Hoffmann et al., 2009).

**TABLE 1:** Adhesion extent scoring (% of defect).

Score	Description
0	0%
1	1–25%
2	26–50%
3	51–75%
4	76–100%

**TABLE 2:** Adhesion strength scoring.

Score	Description
0	No adhesions present
1	Friable
2	Immature, easy to break
3	Mature, hard to break

### Statistical Analyses

A total adhesion score (extent + strength of each defect) was calculated for each animal, with a maximum possible score of 14 points (Tables 1, 2). Adhesion scores were compared between control and experimental animals using the unpaired Student T-test, and the relative incidence of adhesions in the two groups were compared by Fisher Exact test. For both statistical tests, unpaired two-tailed *p*-values <0.05 were considered indicative of statistically significant differences. Data are presented as mean ± SD, or as *n* (%) of group. Animal group size and allocation used a matching ratio (κ) of 1.6 (five Controls and eight Experimentals), which provided >80% power (1-β), with α = Type I error set to 0.05, to detect a 50% difference in mean Total Adhesion Score between groups, using the assumptions of mean Adhesion Scores being 10.0 in Controls and 5.0 in RADA16-treated animals (possible range 0–14) with an estimated SD of 2 and assuming normally distributed data. Data were analyzed using Prism v.5.03 statistical and graphing software (GraphPad Software Inc., San Diego, CA).

## Results

**FIGURE 2:**
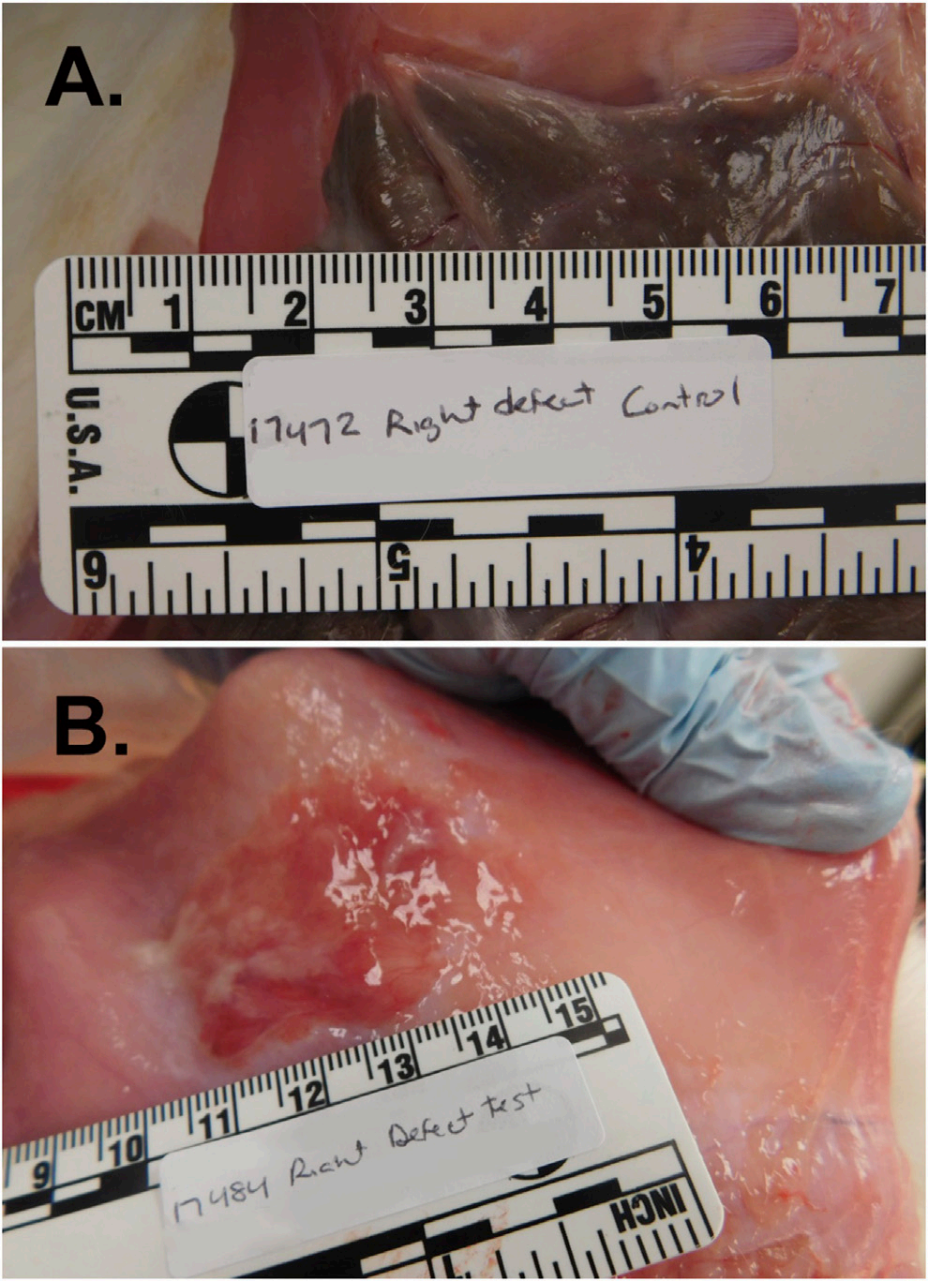
Abdominal adhesion formation at the cecal serosa/peritoneal junction in the rabbit cecal sidewall injury model. This model predictably and quickly generates robust abdominal adhesion formation, with grossly visible adhesions typically present by 7–14 days after surgery. Briefly, the abdominal cavity was accessed through a 12-cm ventral midline incision and bleeding injury was induced on the cecal serosa by abrasion with a sterile dry gauze pad and on the juxtaposed peritoneal inner surface by shallow excision and scraping with a scalpel blade. Approximately ≈2 × 4.5 cm wounds were irrigated with saline and then either treated with topical administration of PuraStat solution or left untreated as Controls before midline incision closure and recovery. At postoperative Day 14, adhesions were significantly more numerous and sizeable at the cecum/peritoneum interface in Control [Panel **(A)**] animals compared to PuraStat-treated animals [Panel **(B)**] whose abraded tissue surfaces received 3–12 ml/lesion of PuraStat solution (2.5% RADA16) immediately after wound creation and just prior to abdominal closure. The mean ± SD Total Adhesion Score (average of the scores for extent + strength of the adhesions in both defects per animal) was significantly 76% lower in PuraStat-treated animals at 2.0 ± 3.0 points compared to 8.2 ± 1.9 points in untreated control animals (*p* = 0.029 by unpaired, two-tailed t-test; maximum possible score = 14 points). The self-assembling RADA16-based hydrogel significantly reduced both the adhesion extent (percent of initial wound area covered by adhesions) and adhesion durability. Remnant PuraStat was observed in 75% of treated animals at the 14-days study terminus.

All animals tolerated the procedure and completed the study protocol. Nine of ten (90%) untreated (control) sidewall defect sites exhibited abdominal adhesions adjoining the abraded cecum and peritoneal wall while 4 of 16 (25%) defect sites treated with PuraStat resulted in adhesion formation (*p* = 0.004 by Fisher Exact test; Table 3). The 90% adhesion formation for the untreated defect sites is consistent with previous results obtained at this laboratory for this model (not shown). RADA16 intervention significantly reduced postoperative adhesion extent and strength. The mean ± SD Total Adhesion Score (average of the values for extent + strength of the adhesion in both defects per animal) was significantly 76% lower in PuraStat-treated animals at 2.0 ± 3.0 points compared to 8.2 ± 1.9 points in untreated control animals (*p* = 0.029; maximum possible score = 14 points; Table 3). Composite adhesion extent values per animal (both lesions), reflecting the percentage of the initial wound area occupied by fibrous adhesions, were 79% lower with PuraStat treatment (mean score 1.0 ± 1.6 points) versus controls (4.8 ± 1.1 points) (*p* < 0.001; maximum possible score = 8 points). Adhesion durability values were similarly and significantly 72% lower in PuraStat-treated animals (1.0 ± 1.5) than in untreated controls (3.6 ± 0.9 points) (*p* = 0.005; maximum possible score = 6 points). Representative photographs of PuraStat-treated and control injury sites are provided in Figure 2 and support the improved lesion site healing with minimal adhesion formation (Table 3). The disparities between the total adhesion scores and subscores in PuraStat-treated versus control animal lesions suggests that PuraStat treatment has a positive effect on preventing adhesion formation at the interface of the injured cecum and overlying peritoneal sidewall defect.

**TABLE 3:** Adhesion scores in abraded cecum, and PuraStat volumes administered.

Group	Animal	Scoring of adhesions to abraded cecum									
		Left side defect				Right side defect					Bilateral total adhesion score
		Test Vol. (ml)	Extent (0 = none)	Strength	Unilateral adhesion score (extent + strength)	Test Vol. (ml)	Extent (0 = none)	Strength		Unilateral adhesion score (extent + strength)	
Control	17,490	—	1	2	3	—	4	2		6	9
	17,486	—	4	2	6	—	2	2		4	10
	17,476	—	2	2	4	—	2	2		4	8
	17,477	—	0	0	0	—	3	2		5	5
	17,472	—	4	2	6	—	1	2		3	9
						Total adhesion score, control, mean					8.2
						Standard deviation					1.9
Test	17,484	6	0	0	0	12	0	0	0		0
	17,485	6	0	0	0	6	0	0	0		0
	17,482	3	4	2	6	3	0	0	0		6
	17,479	3	0	0	0	3	0	0	0		0
	17,470	3	0	0	0	3	0	0	0		0
	17,471	8.5	0	0	0	8	0	0	0		0
	17,471	6	0	0	0	6	1	2	3		3
	17,467	6	1	2	3	6	2	2	4		7
	Wounds with adhesions, control, *n* (%)				9 (90%)	Total adhesion score, test, mean					2.0
	Wounds with adhesions, test, *n* (%)				4 (25%)	Standard deviation					3.0
	*p*-value, two-tailed, Fisher exact test				0.0004	*p*-value, two-tailed, unpaired, student t-test					0.0289

Additional macroscopic observations included the presence of remnant test article (grossly visibly as a clear gelatinous mass) in 6 of 8 (75%) treated animals at the 14-days study terminus. Secondary adhesions of the cecum were observed in all animals and included cecum to: cecum, healing incision site, omentum, large and small intestine, sidewall (without defect), uterus, mesentery, and/or abdominal fat (not shown). Four treated animals (50%) exhibited a tan film on the surface of the liver (*n* = 4) and/or spleen (*n* = 1) that was adjudicated to be probable residual test article because it was not observed in any of the untreated animals.

Total volumes of 3–12 ml PuraStat (as deemed sufficient by the surgeon) were applied to sidewall defect sites after moistening the lesions. Six defect sites received 3 ml of test article and seven sites had 6 ml of test article (Table 3). In three of the 16 wound sites that received PuraStat, the material slipped off the lesion during initial application before complete hydrogel formation had occurred. Additional PuraStat was applied following aspiration of the slipped-off material: one site each received 8, 8.5, and 12 ml of PuraStat in total depending on the amount of slippage of the previous applications. The surgeon’s goal was to achieve, as closely as practicable, a uniform ≈3-mm thick hydrogel layer at the wound interface (3 ml distributed over a 9.0-cm^2^ wound surface area).

Seven of the 13 animals exhibited weight loss of greater than 10% of their body weight on study Day 7, and all but one animal (17,485) increased or maintained body weight from study Day 7 to study terminus on Day 14 (Supplementary Table S1). No animal was excluded by the Study Veterinarian due to weight loss deemed unhealthy.

## Discussion

Our animal study demonstrated that intraoperative administration of PuraStat, a self-assembling RADA16-based hydrogel, can reduce adhesion formation after abdominal surgery. RADA16 (acetyl-[arginyl-alanyl-aspartyl-alanyl]_4_-amide tetrahydrochloride) is a unique synthetic peptide that spontaneously self-assembles to form nanofibers in aqueous environments. The RADA16 nanofibers physically cross-link upon contact with blood and tissue fluids to form a biocompatible hydrogel (Edwards Gayle and Hamley, 2017; Zhang, 2017; Lee et al., 2019; Wang et al., 2019; Sankar et al., 2021). Formulations of RADA16 are used globally as a topical hemostat to stop intraoperative bleeding and prevent postoperative bleeding (United States Food and Drug Administration, 2021; 3-D Matrix Inc. and Needham, 2014). We report here that when applied to the abdominal wounds in the rabbit cecal model, PuraStat adheres to the oozing/bleeding lesion and forms a hemostatic barrier that also reduces the formation of adhesions.

Abdominal adhesions commonly develop after surgery of the abdominal and pelvic cavities (Menzies and Ellis, 1990; Ellis et al., 1999; Okabayashi et al., 2014; Tabibian et al., 2017). While reported rates of postoperative abdominal adhesion development vary across studies, they are nonetheless consistently high. For example, in a postmortem study of 752 individuals, adhesion prevalence was 67% in those with a documented history of prior abdominal surgery (Weibel and Majno, 1973). In a prospective study of 210 patients undergoing a laparotomy who had previously undergone abdominal surgery, 93% reportedly had intra-abdominal adhesions (Menzies and Ellis, 1990). Metabolic disorders associated with aging are thought to increase the likelihood of postoperative adhesion development (Pilpel et al., 2019), so the rate and cumulative societal burden of adhesions will probably increase as the world population gets older. Unlike temporary postoperative complications such as infection and bleeding, adhesions can increase long-term risk of serious health conditions. For example, adhesions are the causative factor of the majority, up to 65%, of the 350,000 small intestine obstruction cases that occur annually in the US alone, acting by compressing or contorting the bowels with adhesive bands of scar tissue (Rami Reddy and Cappell, 2017; ten Broek et al., 2013a). If repeated abdominal or pelvic surgery is needed, even asymptomatic adhesions increase risk by increasing operative time by an average of 15 min during both laparoscopic and open procedures (ten Broek et al., 2013a). Additionally, intra-operative adhesiolysis results in a mean rate of iatrogenic bowel injury in 6% of all repeated abdominopelvic surgeries, including up to 9% of lower GI tract procedures (ten Broek et al., 2013b; Strik et al., 2018), and adhesions are likely to reappear (Tabibian et al., 2017). To date, practical means to reduce postoperative adhesion formation remain largely limited to improving surgical technique to minimize organ surface drying and trauma from excessive handling, and attentiveness to excluding foreign debris such as gauze threads and glove powders from the operative site (Moris et al., 2017). However, these efforts have so far proven insufficient for reducing the likelihood and extent of postoperative abdominal adhesion formation. Because treatment options for established adhesions are few (Hindocha et al., 2015), we tested a new approach for preventing surgically-induced adhesion formation in the first place, using an established animal model (Rodgers et al., 1998; Cooper et al., 2007; Yeo et al., 2007; Hermans et al., 2012). In the rabbit cecal sidewall injury model, intraoperative administration of a biocompatible solution of self-assembling peptides that physically cross-link into a protective hydrogel barrier effectively reduced adhesion formation by more than 70%. Both abdominal adhesion area and strength were similarly and significantly reduced by PuraStat treatment.

A related RADA16-based product is PuraSinus®, cleared by the US FDA in 2019 for use during nasal surgery and after trauma not only as a hemostatic wound dressing, but also as a space-filling gel to separate and prevent adhesion formation between mucosal surfaces, and support wound healing (United States Food and Drug Administration, 2021; 3-D Matrix Europe SAS, 2019). A case series that evaluated RADA16 (as PuraStat) for hemostasis in 60 subjects undergoing endoscopic turbinoplasty also demonstrated no adhesion formation on the endonasal mucosa and normal wound healing in all cases (Lee et al., 2017). The fluid RADA16 formulation was easy to apply using a syringe and catheter and provided full wound coverage. RADA16 was also endonasally administered to a 49-year-old man during endoscopic surgery to divide a severe nasopharyngeal stenosis caused by chemoradiotherapy for oral carcinoma (Wong et al., 2020). Through postoperative 2 months, nasal obstruction remained absent and no adhesion tissue was present. Taken together with our current demonstration that PuraStat can inhibit abdominal adhesion formation after surgical insult, the totality of available evidence indicates that RADA16-based hydrogels may have utility in safely preventing unwanted and inappropriate scar tissue bridging between diverse tissues, and this may be an effective approach for reducing the incidence and extent of adhesions after abdominopelvic surgery.

Abdominal adhesion formation may be indicative of an aberrant wound healing process (Arung et al., 2011; Rodrigues et al., 2019). Appropriate wound healing requires the complex interaction of multiple factors within the wound milieu, including cytokines and growth factors that recruit and activate cellular participants, local thrombogenic signals, fibrinogenic/fibrinolysis balance, hypoxia levels, and the construction of an appropriate extracellular matrix foundation within damaged tissues (Rodrigues et al., 2019; Sankar et al., 2021). Peritoneal damage causes an inflammatory response that includes a thrombogenic component (Mutsaers et al., 2016). In normal healing, a fibrin plug temporarily forms over the damaged mesothelium and is eventually degraded to expose a healed peritoneal surface (Monk et al., 1994; Arung et al., 2011). Adhesion formation reflects impaired local fibrinolysis and persistence of an abundant fibrous matrix. The void-filling RADA16 hydrogel provides temporary physical separation of the recuperating tissues, which may be sufficient to prevent gross tissue-tissue adhesion and allow for regenerative wound healing. *In vitro*, RADA16-based hydrogels provide a structure that supports diverse cell types involved in wound healing and tissue regeneration including macrophage, fibroblasts, and endothelial cells [reviewed in Sankar et al. (2021) and Wang et al. (2019)]. In various animal models, RADA16 has been shown to support epithelial cell repopulation and healing of middle ear mucosal lesions (Akiyama et al., 2013), facilitate repair of periodontal defects (Takeuchi et al., 2016), and provide neomucosal coverage and reduce submucosal damage in resected GI mucosa (Tsiamoulos et al., 2017; Oumrani et al., 2019). In clinical studies, RADA16 treatment has been associated with wound healing of ulcers after undergoing endoscopic submucosal dissection of the stomach (Uraoka et al., 2016), and esophageal and colorectal tissues (Subramaniam et al., 2020), The porous nanofibrillar meshwork of the RADA16 hydrogel might support wound healing by acting as a scaffold that facilitates the integration and interactions of cells necessary for rejuvenating damaged tissue. In the rabbit cecal sidewall injury model, PuraStat treatment was associated with notably superior non-adhesive healing of cecal/peritoneal lesions than occurred with control lesions.

A primary study strength was the use of an established experimental model that allowed uncomplicated assessment of the significant inhibitory effect that PuraStat/RADA16 treatment had on postoperative abdominal adhesion formation. A primary study limitation was that histological or biochemical assessments were not performed; these might elucidate some of the underlying cellular and molecular mechanisms of RADA16-mediated adhesion reduction, such as exploring local expression of biochemical mediators with known roles in wound healing and fibrogenesis. An optimal material barrier against adhesion formation would be easy to apply, biodegradable, non-inflammatory, non-immunoreactive, be retained *in situ* without requiring suturing or other attachments, and function in the presence of blood, RADA16 hydrogels satisfy all these criteria. This animal study demonstrated the potential value of future clinical study of PuraStat/RADA16 as a safe and effective interventional approach for reducing the occurrence of postoperative abdominal adhesions.

## Data Availability

The raw data supporting the conclusion of this article will be made available by the authors, without undue reservation.

## Abbreviations

3D, 3-dimensional; AE, adverse event; RADA16, (arginine-alanine-arginine-aspartic acid)_4_.
