# Supplementary material for: PuraStat RADA16 Self-Assembling Peptide Reduces Postoperative Abdominal Adhesion Formation in a Rabbit Cecal Sidewall Injury Model
Source: Front Bioeng Biotechnol. 2021 Dec 10;9:782224. doi: 10.3389/fbioe.2021.782224 (PMC8703061; doi:10.3389/fbioe.2021.782224)
Supplement: Supplementary file 1 [file Table1.docx]

**Supplemental Files**

Table S1. Animal weights.

| Group | AnimalNumber | Body Weight (kg) | | |
| --- | --- | --- | --- | --- |
|  |  | Pretreatment | Day 7 | Day 14 |
| Control | 17490 | 4.5 | 3.9 | 4.0 |
|  | 17486 | 4.1 | 3.5 | 3.9 |
|  | 17476 | 4.2 | 4.0 | 4.0 |
|  | 17477 | 4.8 | 4.4 | 4.4 |
|  | 17472 | 4.0 | 3.4 | 3.7 |
| Test | 17484 | 4.2 | 3.5 | 3.8 |
|  | 17485 | 4.0 | 3.8 | 3.6 |
|  | 17482 | 4.6 | 3.9 | 3.9 |
|  | 17479 | 4.0 | 3.9 | 3.9 |
|  | 17470 | 4.2 | 3.8 | 3.8 |
|  | 17471 | 4.4 | 3.9 | 4.1 |
|  | 17467 | 4.6 | 4.2 | 4.2 |
|  | 17474 | 4.1 | 3.6 | 3.6 |
